# The roles of sirtuins in ferroptosis

**DOI:** 10.3389/fphys.2023.1131201

**Published:** 2023-04-20

**Authors:** Jieqing Zeng, Junhao Guo, Si Huang, Yisen Cheng, Fei Luo, Xusan Xu, Riling Chen, Guoda Ma, Yajun Wang

**Affiliations:** ^1^ Maternal and Children’s Health Research Institute, Shunde Women and Children’s Hospital, Guangdong Medical University, Foshan, China; ^2^ Institute of Respiratory, Shunde Women and Children’s Hospital, Guangdong Medical University, Foshan, China

**Keywords:** sirtuins, ferroptosis, iron metabolism, lipid peroxidation, reactive oxygen species

## Abstract

Ferroptosis represents a novel non-apoptotic form of regulated cell death that is driven by iron-dependent lipid peroxidation and plays vital roles in various diseases including cardiovascular diseases, neurodegenerative disorders and cancers. Plenty of iron metabolism-related proteins, regulators of lipid peroxidation, and oxidative stress-related molecules are engaged in ferroptosis and can regulate this complex biological process. Sirtuins have broad functional significance and are targets of many drugs in the clinic. Recently, a growing number of studies have revealed that sirtuins can participate in the occurrence of ferroptosis by affecting many aspects such as redox balance, iron metabolism, and lipid metabolism. This article reviewed the studies on the roles of sirtuins in ferroptosis and the related molecular mechanisms, highlighting valuable targets for the prevention and treatment of ferroptosis-associated diseases.

## 1 Introduction

### 1.1 Ferroptosis

Ferroptosis is characterized by fatal lipid reactive oxygen species (ROS) and massive iron-dependent cell death. Before ferroptosis was defined, cell death is classified into apoptosis, autophagy-related cell death, and necrosis according to distinct morphological features (Kroemer et al., 2005). However, Dolma and Yagodac et al. found that cell death induced by RAS-selective lethal (RSL) compounds could not be classified as the traditional form of cell death (Dolma et al., 2003; Yagoda et al., 2007). In 2012, Dixon proposed the concept of ferroptosis based on distinct morphological, biochemical, and genetic features of RSL-induced cell death (Dixon et al., 2012). After that, studies related to ferroptosis is growing exponentially.

**FIGURE 1:**
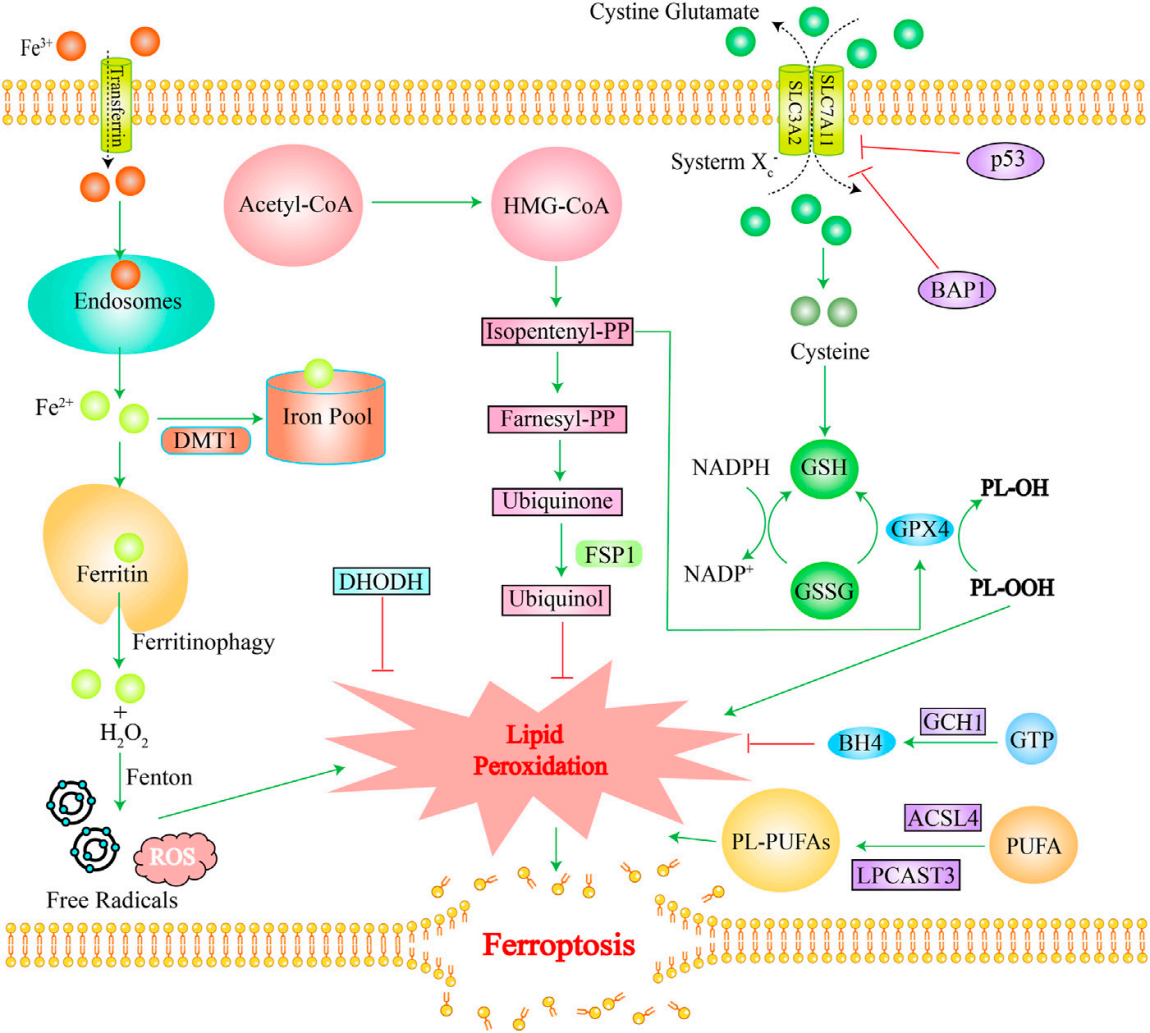
The main mechanism of ferroptosis. Green lines stand for promotion effects and red lines stand for inhibition effects. Iron overload, ROS accumulation, and lipid peroxidation induce ferroptosis. The GPX4 pathway, FS1-Ubiquinol pathway, GCH1-BH4 pathway, and DHODH pathway inhibit ferroptosis. Ferritin releases Fe^2+^ via “ferritinophagy” which initiates a Fenton reaction with H_2_O_2_ produced, generating excessive ROS and free radicals. DHODH detoxifies lipid peroxides and inhibits ferroptosis. FSP1 converts Ubiquinone into Ubiquinol, thereby inhibiting lipid peroxidation. GPX4 requires GSH as a cofactor to promote inhibition of lipid peroxidation. The system Xc^—^exchanges cystine and glutamic acid, which are converted to cysteine for glutathione synthesis. BAPI and p53 genes can inhibit cystine uptake by inhibiting the expression of SLC7A11. GCH1 catalyzes GTP to produce BH4, reshaping lipids to inhibit ferroptosis. ACSL4 and LPCAT3 are necessary for production of PL-PUFAs, which promote ferroptosis. Abbreviation: DMT1, divalent metal transporter 1; Acetyl-CoA, Acetyl-coenzyme A; FSP1, ferroptosis suppressor protein 1; BAP1, BRCA1 associated protein 1; GSH, glutathione; GSSG, Oxidized glutathione; BH4, Tetrahydrobiopterin; GCH1, GTP cyclohydrolase-1; DHODH, dihydrolactate dehydrogenase; GTP, guanosine triphosphate cyclohydrolase-1; ACSL4, long-chain fatty acyl-CoA; LPCAST3, lysolecithin acyltransferase 3; HMG-CoA, 3-Hydroxy-3-Methyl-Glutaryl-CoA; PP, diphosphate.

Ferroptosis mainly involves three mechanisms. The first one is an increased intracellular free iron content: extracellular Fe^3+^ is transported into intracellular endosomes through transferrin and reduced to Fe^2+^, which is released into the cytoplasmic iron pool via the divalent metal-ion transporter 1 (DMT1), while the excess iron is stored in ferritin (Frazer and Anderson, 2014; Xie et al., 2016). Under some circumstances, ferritin releases Fe^2+^ via “ferritinophagy” which initiates a Fenton reaction with the NADPH oxidase NOX, or with H_2_O_2_ produced by the mitochondrial electron transport chain, generating excessive ROS and free radicals (Jiang et al., 2021). The second one is the impaired glutathione peroxidase 4 (GPX4) activity: phospholipid hydroperoxides (PLOOHs) are executioners of ferroptosis. GPX4 is the major enzyme that catalyzes the reduction of PLOOHs in mammals (Yang et al., 2014). Generally, GPX4 requires two electrons from GSH for the reduction of phospholipid and cholesteryl hydroperoxides to their corresponding alcohols, thereby reducing PLOOHs; the blockade of the antioxidant system, cystine-glutamate antiporter (system Xc^−^), can lead to insufficient glutathione (GSH) synthesis. The system Xc^−^ consists of two subunits (SLC7A11 and SLC3A2) that transport cystines into cells and glutamate out of the cells simultaneously. Cystine is a dimer of cysteine (Murphy et al., 1989). Cysteine is an important component in the synthesis of GSH and its deficiency will cause insufficient cellular GSH synthesis, which will affect the function of GPX4, ultimately leading to ferroptosis (Stockwell et al., 2017; Hassannia et al., 2019). The third important one is lipid peroxidation: polyunsaturated fatty acids (PUFA) are important initiators of lipid peroxidation (Jiang et al., 2021). Phospholipids containing polyunsaturated acyl tails (PL-PUFAs), activated by enzymes such as long-chain fatty acyl-CoA synthetase 4 (ACSL4) and lysolecithin acyltransferase, mainly promote lipid peroxidation (Gill and Valivety, 1997). In the final stage of ferroptosis, lipid peroxidation directly or indirectly induces the formation of pores in the cell membrane, which triggers cell death (Kagan et al., 2017). Ferroptosis has its unique characteristics. Morphologically, ferroptosis is characterized mainly by dysfunction of mitochondria as excess iron drives the peroxidation of plasma-membrane lipids and affects the fluidity and integrity of the plasma membrane, which results in the rupture of the outer mitochondrial membrane, shrinkage, cristae reduction, and even disappearance, and ultimately disrupts mitochondrial function (Jiang et al., 2021). Metabolically, in addition to increased intracellular iron, ferrous ions, and ROS, ferroptosis is often accompanied by reduced GSH metabolism and other changes (Zhang et al., 2022a). More specific mechanisms of ferroptosis have been reported in several studies (Dixon et al., 2012; Hadian and Stockwell, 2020; Jiang et al., 2021; Qi et al., 2022; Stockwell, 2022). Figure 1 shows the main mechanism of ferroptosis.

Ferroptosis plays a significant role in tumors, neurodegenerative diseases, cardiovascular and cerebrovascular diseases, metabolic diseases, and respiratory diseases (Galaris et al., 2006; Dixon et al., 2012; Heemels, 2016; El Hout et al., 2018; Ma et al., 2019; Yoshida et al., 2019; Wang et al., 2021a; Cheng et al., 2021; Chen et al., 2022a). For instance, in neoplastic diseases, high ROS levels and high iron contents in cancer cells render them more susceptible to ferroptosis than normal cells due to their high metabolism (Dixon et al., 2012; Sosa et al., 2013; Spangler et al., 2016). In neurodegenerative diseases, regional redistribution of iron in brain tissue leads to ferroptosis in portions of the brain tissue (Yan and Zhang, 2019). Ferroptosis induced by intracellular lipid peroxidation and iron accumulation in mitochondria may be an important cause of cardiovascular and cerebrovascular disease since blood circulation is essential for iron transportation (Wortmann et al., 2013; Zhang et al., 2022b). In metabolic diseases such as diabetes, cells in a hyperglycemic environment will produce excess ROS, which can easily cause lipid metabolism disorders and further lead to ferroptosis (Yung et al., 2016; Han et al., 2020). In COVID-19, the SARS-CoV-2 virus can cause cellular iron uptake, depletion of the GSH-GPX4 axis, and ROS overproduction, which all contribute to the Fenton reaction to generate excess lipid hydroperoxides and hydroxyl radicals, ultimately accelerating ferroptosis (Fratta Pasini et al., 2021). Currently, studies have indicated that iron chelators as well as anti-lipid peroxidation drugs may suppress or ameliorate these diseases (Hanson et al., 2009; Dare et al., 2015). It's worth noting that multiple endogenous antioxidant defense systems in the cells can regulate ferroptosis. Notably, the sirtuins family can regulate ferroptosis via mediating multiple target genes and is a potential target for the treatment of ferroptosis-related diseases.

### 1.2 Sirtuins family

In 1979, Amar Klar identified a protein (mating-type regulator, MAR1) that could silence a gene locus in yeast (Klar et al., 1979). Then, three proteins with similar functions were found by other investigators (Tissenbaum and Guarente, 2001; Rogina and Helfand, 2004; Viswanathan and Guarente, 2011), all of which were uniformly named sirtuins (Frye, 2000). Among them, MAR1 was designated as Sir2 (Grunstein, 1997). In 1999, Kaeberlein et al. unveiled that sirtuins could significantly extend yeast lifespan by 30% (Kaeberlein et al., 1999), which attracted much attention. Mammal sirtuins comprise seven isozymes (SIRT1-SIRT7). Based on molecular genetic analysis of different biological types and domain sequences, the seven sirtuins can be classified into four distinct classes (Frye, 2000; Greiss and Gartner, 2009; Donmez and Guarente, 2010; Cen et al., 2011; Teixeira et al., 2020): class I (SIRT1, 2, and 3), class II (SIRT4), class III (SIRT5), and class IV (SIRT6 and SIRT7) (Roth et al., 2013). Sirtuins are all composed of a small domain (a Zn^2+^ domain consisting of about 40 amino acids) and a large domain (a Rossman fold consisting of about 200 amino acids) (Chang and Guarente, 2014). Only when NAD^+^ and acetyl-lysine can enter the active site from either end of the cleft between these two domains, the molecular conformation of the sirtuins can be stretched and extended to activate the function of deacetylases and ADP ribosyltransferases (Bordo, 2013).

Sirtuins are differentially expressed in tissues and organs, and the tissues and organs with high expression of sirtuins members are summarized in Table 1. Collectively, sirtuins are mainly highly expressed in tissues, organs, and embryos with high metabolic rates. Additionally, sirtuins also have different cellular localizations and functions in cells. SIRT1, SIRT6, and SIRT7 are mainly located in the nucleus, SIRT3, SIRT4, and SIRT5 are mainly situated in the mitochondria, whereas, only SIRT2 is mainly presented in the cytoplasm (Vaquero, 2009; Houtkooper et al., 2012; Wang and Lin, 2021). Notably, the subcellular localization of these sirtuins also depends on the state of the cell as well as molecular interactions; for example, SIRT1 and SIRT2 can translocate between the nucleus and the cytoplasm, and SIRT3 can shuttle between mitochondria and the nucleus, where they can interact with proteins (Houtkooper et al., 2012; Bonkowski and Sinclair, 2016). The sirtuins family acts as an NAD^+^-dependent histone deacetylase and the function is linked to deacetylation and ADP-ribosyltransferase activity. SIRT1, SIRT2, and SIRT3 have strong histone deacetylase activity, whereas, SIRT4-SIRT7 have weak deacetylase activity (Vaquero, 2009). In addition, SIRT4 and SIRT6 possess ADP-ribosyltransferase activity (Chen et al., 2015). They can modulate multiple pathways such as glucose and fatty acid metabolism, anti-aging, apoptosis, DNA repair, neuronal production, inflammatory responses, and even the regulation of the circadian clock by regulating post-translational modifications of histones and transcription factors (Haigis and Sinclair, 2010; Chang and Guarente, 2014).

**TABLE 1:** Molecular structure, enzymatic activity, regulators, localization, and tissue expression patterns of sirtuins.

Classes		Enzymatic activity	Inhibitors	Activators	Primary locallization	High expressed organs
I	SIRT1 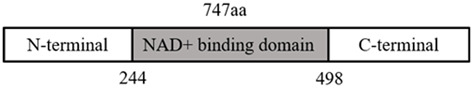	deacetylase	EX527 (Dai et al., 2018), Sirtinol (Grozinger et al., 2001), Suramin (Trapp et al., 2007), Tenovin (Botta et al., 2012), Salermide (Lara et al., 2009), Compound 15e (Sundriyal et al., 2017), SirReal2 (Eid et al., 2022), ELT-11c (Disch et al., 2013), Compound 8 (Mahajan et al., 2014), Compound 28e (Yang et al., 2017b), UBCS0137 (Sundriyal et al., 2017), 3′-(3-fluoro-phenethyloxy)-2-anilinobenzamide (Suzuki et al., 2012)	SRT2104 (Milne et al., 2007), Resveratrol (Dai et al., 2018), SRT1720 (Milne et al., 2007), SRT3657 (Gräff et al., 2013), SRT1460 (Pacholec et al., 2010), SRT2183 (Pacholec et al., 2010), piceatannol (Gertz et al., 2012), 1,4-DHP derivative (Mai et al., 2009), Ainsliadimer C (Chen et al., 2022c), Scopolin (Yoo et al., 2017), Tenovin-6 (Ladds et al., 2020), CAY10602 (Nayagam et al., 2006), YK-3-237 (Ponnusamy et al., 2015), Nicotinamide riboside (Ponnusamy et al., 2015), Agrimol B (Hnit et al., 2021), Butein (Zhang et al., 2015), Ophiopogonin D' (Wang et al., 2017a)	nuclear	B, E, H, K, Lu, O, SM, U Chang and Guarente, (2014)
	SIRT2 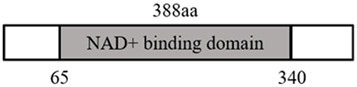	deacetylase	AGK2 (Outeiro et al., 2007), Sirtinol (Grozinger et al., 2001), ELT-11c (Disch et al., 2013), Thiomyristoyl (Jing et al., 2016), SirReal2 (Rumpf et al., 2015a), Salermide (Lara et al., 2009), UBCS0137 (Sundriyal et al., 2017), Compound 15e (Sundriyal et al., 2017), Compound 28e (Yang et al., 2017b), Compound 8 (Mahajan et al., 2014), 3′-(3-fluoro-phenethyloxy)-2-anilinobenzamide (Suzuki et al., 2012)	SRT1720(65), 1,4-DHP derivative (Mai et al., 2009)	cytosol	B, E, Li, Lu, P, SM Gomes et al., (2015)
	SIRT3 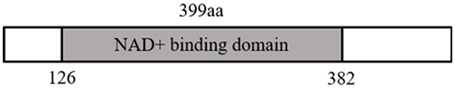	deacetylase	SirReal2 (Rumpf et al., 2015a), Compound 15e (Sundriyal et al., 2017), CHIC35 (Rumpf et al., 2015b), UBCS0137 (Sundriyal et al., 2017), ELT-11c (Disch et al., 2013), Compound 28e (Yang et al., 2017b), Compound 8 (Mahajan et al., 2014)	3-TYP (Pi et al., 2015),CrocinⅠ (Xiao et al., 2019), piceatannol (Gertz et al., 2012), Resveratrol (Dai et al., 2018), 1,4-DHP derivative (Mai et al., 2009)	mitochondria	H, E, Li, SM Shi et al., (2005)
II	SIRT4 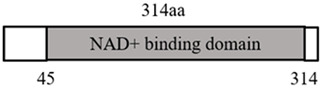	deacetylase & ADP-ribosylation	Tunicamycin (Akkulak and Yalcin, 2022), SirReal2 (Rumpf et al., 2015a)	_	mitochondria	B, E, H, K, Li (Haigis et al., 2006; Mathias et al., 2014)
III	SIRT5 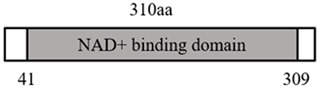	deacetylase	Suramin (Wang et al., 2017b), NRD167 (Li and Melnick, 2021),	MC3138 (Hu et al., 2021), piceatannol (Gertz et al., 2012), Resveratrol (Dai et al., 2018), UBCS039(64)	mitochondria	B, E, H, K, Li, Th Rardin et al., (2013) Nakamura et al., (2008); Park et al., (2013); Kumar and Lombard, (2018)
			MC3482 (Polletta et al., 2015), SirReal2 (Rumpf et al., 2015a), UBCS0137 (Sundriyal et al., 2017)			
IV	SIRT6 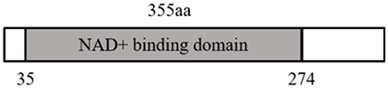	deacetylase & ADP-ribosylation	Compound 1 (Dai et al., 2018), SirReal2 (Rumpf et al., 2015a), OSS_128167 (Zou et al., 2021)	UBCS039(64)	nuclear	B, E, H, K, SM Korotkov et al., (2021)
	SIRT7 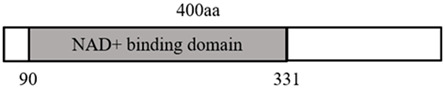	deacetylase	SIRT7 inhibitor 97491 (Kim et al., 2019)	_	nuclear	E, Li, Te Li et al., (2019)

Abbreviation: B, brain; E, embryon; H, heart; K, kidney; Li, Liver; Lu, Lung; O, ovary; P, pancreatic; SM, skeletal muscle; Te, Testicles; Th, Thymus; U, uterus.

Sirtuins have been identified as therapeutic targets for many diseases (such as tumors, cardiovascular diseases, and nervous system diseases) (Ford et al., 2005; Ajami et al., 2017; Kane and Sinclair, 2018; Manjula et al., 2020), so the regulators of sirtuins are research hotspots in recent years. For example, Selisistat (EX 527, SEN0014196) is currently the only SIRT1-targeted drug in the stages of clinical trials for the treatment of Huntington’s disease (Rollnik, 2015). Selisistat can occupy the nicotinamide binding site and the c-pocket adjacent to nicotinamide and bind to NAD^+^ ribose or the coproduct 2′-O-acetyl-ADP-ribose, showing a strong inhibitory effect on SIRT1 and weak inhibitory effects on SIRT2, SIRT3, and SRT6 but no effect on SIRT5 (Napper et al., 2005; Gertz et al., 2013). Inhibitors of sirtuins effectively induce cellular and physiological effects through diverse substrate sites (Rajabi et al., 2018), while activators of sirtuins only require an allosteric binding site, and, therefore activators have stronger targeting specificity and fewer side effects (Dai et al., 2018). A synthetic sirtuin-activating compound with imidazothiazole as its core can potently activate SIRT1 (Milne et al., 2007). For example, there are currently 14 clinical trials (www.clinicaltrials.gov) for SRT2104 showing beneficial effects (Baksi et al., 2014; van der Meer et al., 2015; Sands et al., 2016). The classification, molecular structure, function, inhibitors, activators, and subcellular localization of mammalian sirtuins are listed in Table 1.

The sirtuins family not only can effectively reduce ROS, suppress inflammation, and decrease lipid peroxide and iron levels but also can protect against or alleviate ferroptosis. At present, the development and gradual maturing of activators and inhibitors of sirtuins provide a powerful tool for investigating the roles of sirtuins in ferroptosis.

## 2 Roles of sirtuins in ferroptosis

### 2.1 SIRT1

SIRT1, the earliest and most intensively studied sirtuin family member of class III deacetylases, possesses potent deacetylase activity and is widely involved in multiple biological processes including aging, apoptosis, autophagy, stress, inflammation, and energy metabolism (Guo et al., 2015; Mu et al., 2015; Li et al., 2016). SIRT1 has recently been demonstrated to regulate ferroptosis via mediating multiple target genes.

P53: SIRT1 can deacetylate p53 to suppress ferroptosis. P53 can regulate ferroptosis through pathways dependent or independent of GPX4 (Espinosa-Diez et al., 2015; Chen et al., 2021). On the one hand, p53 can induce ferroptosis by directly inhibiting the GPX4 pathway (Liu et al., 2021a). On the other hand, under ROS stress, p53 can either inhibit cystine uptake by repressing SLC7A11 expression or enhance cellular ferroptosis via indirectly strengthening the function of the lipoxygenase (ALOX) family (Jiang et al., 2015; Chu et al., 2019). Doxorubicin (Dox) has been proven to induce ferroptosis in cardiac cells by initiating the Fenton reaction, producing large amounts of ROS as well as reducing the levels of antioxidant substances (GPX4, SOD, GSH, etc.) (Zhang et al., 2022b). De et al. found that overexpression of SIRT1 repressed the acetylation of p53 and decreased the ROS level in DOX-induced cardiac cells (De Angelis et al., 2015). In light of this finding, we speculated that SIRT1 could suppress DOX-elicited ferroptosis in cardiac cells by deacetylating p53, which was demonstrated by the following evidence. Ma et al. revealed that SIRT1 rescued the suppressive effect of p53 on SLC7A11 by reducing the acetylation level of p53, thereby inhibiting ferroptosis evoked by myocardial ischemia/reperfusion (Ma et al., 2020). Furthermore, SIRT1 has been demonstrated to repress p53 acetylation at the K382 site and the expression level of ACSL4 as well as inhibit lipid peroxidation and the level of malondialdehyde (MDA), a product of lipid peroxidation, while increasing the expression of GPX4 and SLC7A11, thereby alleviating acute lung injury caused by heat stress by inhibiting ferroptosis (Chen et al., 2022b).

Nuclear factor erythroid2-related factor 2 (NRF2): SIRT1 can also exert anti-ferroptosis effects by activating NRF2, a central regulator of the antioxidant stress response (Ma, 2013; Bellezza et al., 2018). NRF2 also acts as a master regulator of iron metabolism and ferroptosis, and most of the genes related to ferroptosis identified to date are target genes of NRF2 (Dodson et al., 2019; Anandhan et al., 2020). Firstly, NRF2 can’t only upregulate ferritin heavy chain 1 (FTH1) and ferritin light chain (FTL) expression to increase the free iron storage in ferritin but also reduce the intracellular iron content and inhibit cellular free iron accumulation via inducing ferroportin (FPN), the only membrane iron exporter (Pietsch et al., 2003; Harada et al., 2011; Kerins and Ooi, 2018). Secondly, NRF2 prevents lipid peroxidation by activating the transcription of the GPX4 gene and activating the expression of the genes involved in the GSH biosynthetic pathway (catalytic and regulatory subunit of glutamate-cysteine ligase, glutathione reductase, SLC7A11), as well as the genes that can promote the reduction of oxidized glutathione (GSSG) to GSH (glucose 6-phosphate dehydrogenase, 6-phosphogluconate dehydrogenase, malic enzyme) (Lu, 2009; Shin et al., 2018; Dodson et al., 2019). These findings collectively illustrate that NRF2 can effectively inhibit ferroptosis. The suppressive effect of SIRT1-NRF2 on ferroptosis has been validated in a variety of diseases. Fisetin exerts its therapeutic effects on DOX-induced cardiomyopathy by inhibiting ferroptosis via SIRT1/NRF2 pathway activation in the rat model of DOX-induced cardiomyopathy and H9c2 cells (Li et al., 2021). Tang et al. also demonstrated that miR-138-5p could exert anti-ferroptosis effects by activating the SIRT1/NRF2 pathway, thereby alleviating diabetic retinopathy (Tang et al., 2022). Zeng et al. demonstrated that resveratrol can alleviate sepsis-induced cardiomyopathy by activating SIRT1 and upregulating the expression of NRF2 to against ferroptosis (Zeng et al., 2023). Wang et al. elucidated that Ulinastatin blocks ferroptosis in liver injury triggered by acetaminophen overdose via the SIRT1-NRF2-HO-1 pathway (Wang et al., 2021b). Also, the latest study has illustrated that mesenchymal stem cell (MSC)-derived exosomes can inhibit hippocampal ferroptosis to alleviate delayed neurocognitive recovery in aged mice via activating the SIRT1/NRF2/HO-1 pathway, while inhibition of SIRT1 activity can abolish the protective effect exerted by MSC-derived exosomes (Liu et al., 2022a). Furthermore, Dang et al. unraveled that Edaravone alleviated ferroptosis in depression and anxiety-like behaviors through the SIRT1/NRF2/HO-1/GPX-4 pathway (Dang et al., 2022). Peroxisome proliferator-activated receptor gamma coactivator 1α (PGC-1α) can regulate mitochondrial biogenesis, upregulate the expression of antioxidant enzymes, and decrease the levels of ROS (Krishnan et al., 2012). NRF2 can regulate PGC-1α to modulate ferroptosis (Miao et al., 2022). Additionally, SIRT1 can also directly activate PGC-1α. SIRT1 can interact with PGC-1α at specific lysine residues and deacetylate it in a NAD (+)-dependent manner, whereby activating the transcriptional activity of PGC-1α, promoting mitochondriogenesis, and suppressing oxidative stress (Rodgers et al., 2005; Baur et al., 2006). The anti-diabetic drug canagliflozin (Cana) potentiates the activity of SIRT1 and AMPK and their downstream target PGC-1α and promotes oxidative phosphorylation in adipocytes, thereby reducing insulin resistance (Yang et al., 2020).

Liang et al. (2023) showed that mitochondrial damage resulting from SIRT1 inactivation played an important role in ferroptosis caused by Epothilone B in schwann cells. Liu et al. found identified that pumilio 2 (PUM2) promoted ferroptosis by inhibiting SIRT1/SLC7A11, aggravated neuroinflammation and brain damage induced by ischemia-reperfusion injury (Liu et al., 2023). In addition, activation of SIRT1 and GPX4 by calorie restriction protects against the ferroptosis-caused kidney injury in the Sprague Dawley rat model of contrast-induced nephropathy (Fang et al., 2021). Ferritinophagy is a form of cell-selective autophagy mediated by nuclear receptor coactivator 4 (NCOA4), which degrades ferritin (mainly FTH1) in autophagosomes, leading to the release of free iron from ferritin-bound iron (Santana-Codina et al., 2021). Ferritinophagy has been linked to ferroptosis (Zhou et al., 2020a). Autophagy has also been proven to promote ferroptosis through the removal of ferritin (Hou et al., 2016). The SIRT1-autophagy axis can protect foam cells from ferroptosis caused by inflammatory cytokines (Su et al., 2021). Recently, some scholars also inferred that the SIRT1-autophagy axis could inhibit oxidative stress-induced ferroptosis in nucleus pulposus cells (Zhou and Ruan, 2022). Silencing miR-34a, a target repressor of SIRT1, can increase SIRT1 expression and decrease hepatic triglyceride accumulation and lipid ROS production in the presence of iron (Wang et al., 2020). Hao et al. reported that treatment of rat pheochromocytoma cells (PC12 cells) with metal cadmium led to ferroptosis by up-regulating miR-34a-5p expression and inhibiting SIRT1 expression (Hao et al., 2022).

In addition to the evidence of direct involvement in ferroptosis, the role of SIRT1 in iron overload and resistance to oxidative stress also suggests its potential mechanisms affecting ferroptosis. Transcription factors of the forkhead box class O (FOXO) family can regulate a variety of genes involved in antioxidant defense (Klotz et al., 2015). FOXO1 has been demonstrated to inhibit lipid accumulation (Kitakata et al., 2021). Iron overload can result in a reduction in the expression of SIRT1 and an increase in acetylated FOXO1 in the nucleus as well as an increased level of oxidative stress. However, FOXO1 acetylation can be reversed when SIRT1 is overexpressed, initiating antioxidant functions and protecting against liver damage from ferroptosis (Das et al., 2016). In addition, studies showed that, by the inhibition of the activity of FOXO1, insulin suppresses gluconeogenesis in the liver and decreases blood glucose, thereby alleviating diabetes (Puigserver et al., 2003; Semova et al., 2022). In diabetes, pancreatic β-cell cells undergo ferroptosis (Bao et al., 2023). We therefore speculate that insulin may regulate FOXO1 by activating SIRT1 to alleviate ferroptosis. FOXO3a can induce antioxidant responses by regulating manganese superoxide dismutase (MnSOD, SOD2) and catalase (CAT) and also regulate mitochondrial activity by inhibiting the c-myc function, thereby blocking the hypoxia-dependent increase in the ROS level (Ferber et al., 2012). SIRT1 exerts its anti-oxidative function in 293T cells under conditions of oxidative stress by interacting with FOXO3a (Brunet et al., 2004). Moreover, SRT2104, as an activator of SIRT1, decreases the synthesis of 4-hydroxynonenal (4-HNE), a marker of lipid peroxides, in the liver and muscle and decreases MnSOD levels in muscle (Mercken et al., 2014).

SIRT1 can repress ferroptosis in cells in the vast majority of cases, but several pieces of negative evidence have also been reported. For example, Zhou et al. (2020b) found that intestine-specific SIRT1 knockout reduces the iron accumulation level in ethanol-induced liver injury to protect against ethanol-induced ferroptosis in hepatocytes. Based on this study, we speculated that SIRT1 knockout could lead to a decrease in iron uptake by intestinal epithelial cells, thereby reducing iron content in the liver and ultimately alleviating ferroptosis in hepatocytes. Moreover, Lee and Sui et al. also revealed that inhibition or silencing of SIRT1 antagonized ferroptosis in head and neck cancer and human papillary carcinoma cells. In other words, SIRT1 overexpression could result in ferroptosis in head and neck cancer and human papillary carcinoma cells, thus preventing tumor progression (Sui et al., 2019; Lee et al., 2020), which might be attributed to the mechanism that SIRT1 overexpression affected cellular homeostasis. When Wang et al. (2023) studied the damage of fluorine to the liver in chicken, they found that sodium fluoride upregulated SIRT1 and triggered the production of ferroptosis. Taken together, SIRT1 can regulate the progression of ferroptosis through multiple pathways and is a valuable potential target for the treatment of ferroptosis-related diseases.

### 2.2 SIRT2

SIRT2 can deacetylate more than 30 substrate proteins and exert regulatory functions in metabolism, lipogenesis, cell cycle progression, oxidative stress, inflammation, etc (Lee et al., 2019). Additionally, SIRT2 can regulate ferroptosis. In a rat model of neuropathic damage, Zhang et al. found that SIRT2 upregulated the expression of FPN1 and GPX4 and downregulated that of ACSL4, leading to reduced iron contents and lipid peroxidation levels, thus alleviating ferroptosis (Zhang et al., 2022c). Gao et al. (2021) demonstrated that in a mouse model of traumatic brain injury, SIRT2 suppressed ferroptosis following traumatic brain injury by decreasing the acetylation level of p53.

Oxidative stress induces massive generation of ROS that damage proteins, lipids as well as nucleic acids, which is an important process in ferroptosis. Studies have shown that the deacetylation function of SIRT2 plays a vital role in redox balance, suggesting that SIRT2 may modulate ferroptosis. Similar to SIRT1, SIRT2 can deacetylate PGC1-α (Krishnan et al., 2012) and FOXO3a (Chen et al., 2013) to upregulate the expression of antioxidant enzymes and downregulate the levels of ROS, thereby alleviating cell injury. NADPH is an important molecule that counteracts oxidative damage by maintaining the reduced-form glutathione. SIRT2 can also deacetylate and activate 6-phosphogluconate dehydrogenase, a key enzyme of the pentose-phosphate pathway, thereby increasing cytosolic NADPH levels and reducing cellular oxidative damage (Wang et al., 2014). Cao et al. (2016) unveiled that SIRT2 inhibitors inhibited Akt phosphorylation and decreased nuclear NRF2 levels, which in turn decreased cellular glutamate cysteine ligase (GCL) and GSH levels, attenuating cellular antioxidant capacity. Yang et al. found that SIRT2 decreased the total and nuclear NRF2 activity and expression via binding to and deacetylating NRF2 on lysines 506 and 508 sites, while a decrease in nuclear NRF2 levels further decreased FPN1 expression and finally reduced the cellular iron export (Yang et al., 2017a). In iron-deficient hepatocytes, this improving effect of SIRT2 on cellular iron level contributes to cell survival. However, whether SIRT2 can elevate cellular iron levels in the presence of high iron levels requires further investigation.

### 2.3 SIRT3

Although all sirtuins can extend the lifespan of yeast, a linkage disequilibrium analysis in 710 subjects has suggested that only SIRT3 correlates with the human lifespan (Bellizzi et al., 2007). SIRT3 is localized in mitochondria, but under cellular stress conditions, such as various stimuli (ultraviolet irradiation and chemotherapy), SIRT3 can transfer from mitochondria into the nucleus to exert NAD⁺-dependent deacetylation function (Alqarni et al., 2021). SIRT3 also exerts crucial roles in the electron transport chain, fatty acid oxidation, amino acid metabolism, iron metabolism, redox balance, and the tricarboxylic acid (TCA) cycle (Finley and Haigis, 2012), by which it regulates the activities of specific metabolic enzymes as well as ATP synthesis, metabolism, and intracellular signaling to relieve oxidative stress (Michishita et al., 2005). Additionally, SIRT3 can regulate mitochondrial disorders, such as cancer, sleep disorders, and Alzheimer’s disease (Lee et al., 2018; Lin et al., 2020; Ouyang et al., 2022). Studies have shown that SIRT3 can regulate diseases by inhibiting ferroptosis.

In the nervous system, ferroptosis is a major contributor to oligodendrocyte (OL) death triggered by glutamate, while inhibition of SIRT3 promotes glutamate-induced ferroptosis in OLs and consequently decreases cell survival, which is critical to the pathobiology of stroke and traumatic brain injury (Novgorodov et al., 2018). The ketogenic diet (KD) is famous for its neuroprotective effects. Compared with the long-chain triglyceride-enriched KD (LKD), the medium-chain triglyceride-enriched KD (MKD) has a stronger preventive effect against the cognitive deficits elicited by sleep deprivation, which may be associated with MKD-caused higher SIRT3 protein levels and inhibition of ferroptosis (Wang et al., 2022).

The role of SIRT3 in ferroptosis seems paradoxical in tumors. Cancer cells have vigorous metabolism, and mitochondria are the cellular power plant. It has been illustrated that SIRT3 colocalizes with p53 in mitochondria and deacetylates p53 (Novgorodov et al., 2018). Hence, the anti-ferroptosis effect of SIRT3 in tumor cells potentially functions in the mitochondria. SIRT3 has been also substantiated to suppress p53-mediated ferroptosis under ROS stress stimulation in human osteosarcoma and melanoma cells (Jin et al., 2021). Conversely, in the presence of high glucose concentrations and ferroptosis-inducing compounds, SIRT3 deficiency can disrupt the AMPK-mTOR pathway and increase GPX4 expression to suppress ferroptosis in trophoblasts (Han et al., 2020). Intriguingly, SIRT3 also plays an opposing role in gallbladder cancer. Liu et al. unraveled that SIRT3 silencing could inhibit Akt-dependent death in gallbladder cancer cell lines; *vice versa*, overexpression of SIRT3 boosted ferroptosis in gallbladder cancer cells and repressed tumor initiation and progression (Liu et al., 2021b). P53 is well-known as a very important target of SIRT3, and p53 mutation or loss-of-function frequently occurs in tumor cells, but neither of the latter two studies assessed the function of p53, which may be one of the reasons for the aforementioned contradictory effects of SIRT3 on ferroptosis.

In the heart, the angiotensin receptor enkephalin inhibitor LCZ696 increased the expression of SIRT3 and deacetylated its target gene SOD2 by activating AKT, thereby inhibiting ferroptosis, and ultimately preventing cardiac toxicity caused by DOX (Liu et al., 2022b). In the respiratory system, cigarette smoke extract inactivated the NRF2/SIRT3 signaling pathway through ROS, thereby promoting the expression of iNOS, ultimately leading to ferroptosis in bronchial epithelial cells (Zi et al., 2023). In the reproductive system, metformin can improve polycystic ovary syndrome by upregulating the expression level of SIRT3 and activating the SIRT3/AMPK/mTOR pathway to inhibit ferroptosis (Peng et al., 2023).

Iron overload, ROS production, and lipid peroxidation are important factors in the development of ferroptosis. Iron regulatory proteins (IRPs) are implicated in the maintenance of cellular iron homeostasis and can regulate the expression of iron metabolism-associated genes by binding to the iron-responsive element (IRE) of target mRNAs (Pantopoulos, 2004). Mitochondrial SIRT3 regulates cellular iron metabolism via controlling the IRP1 activity, while SIRT3 deficiency leads to an imbalance of cellular iron homeostasis. SIRT3 overexpression decreases the expression of TfR1 (a membrane-associated glycoprotein critical for iron uptake and cell proliferation) and suppresses pancreatic cancer cell proliferation by repressing IRP1 (Jeong et al., 2015). In addition, the knockdown of SIRT3 can exacerbate iron overload and NADPH oxidase-derived ROS production while increasing the levels of acetylated p53, HO-1, and FPN (Feng et al., 2020). It has been evidenced that SIRT3 regulates iron overload-induced hepatocyte death through the Wnt/β-catenin pathway (Mandala et al., 2021). SIRT3 contributes to the maintenance of GSH in the reduced and activated state. SIRT3 converts O^2−^ to H_2_O_2_ as well as NADP^+^ to the TCA cycle of NADPH and also deacetylates isocitrate dehydrogenase 2 (IDH2) and MnSOD, thereby maintaining the oxidative balance (Qiu et al., 2010; Tao et al., 2010; Shi et al., 2017; Sidorova-Darmos et al., 2018). Angiotensin-receptor blocker LCZ696 can suppress DOX-caused cardiotoxicity by activating the AKT/SIRT3/SOD2 pathway and protecting against ferroptosis (Liu et al., 2022b). Besides, SIRT3 can activate NRF2, a key regulator of ferroptosis, stimulating the transcription of downstream antioxidant genes (Gao et al., 2018). Reversely, NRF2 also regulates SIRT3 expression and reduces ROS levels in neuronal cells (Gao et al., 2018).

Altogether, SIRT3 can mediate ferroptosis through multiple pathways in either the mitochondria or the nucleus. However, SIRT3 shows different regulatory roles in ferroptosis in different cells and under different stress conditions, which requires additional in-depth investigation.

### 2.4 Other sirtuins family members

There is currently no definite evidence that SIRT4 can affect ferroptosis, but SIRT4 can compensate for each other with SIRT1 and SIRT3. SIRT4 knockdown in primary hepatocytes increases SIRT1 expression (Nasrin et al., 2010), whereas, SIRT4 overexpression in mice with Angiotensin II-induced cardiac hypertrophy inhibits the binding of MnSOD to mitochondrial SIRT3 and increases MnSOD acetylation levels to reduce its activity, resulting in increased ROS accumulation upon Ang II stimulation (Luo et al., 2017). There is also no evidence for a direct effect of SIRT5 on ferroptosis, but SIRT5 can also suppress the progression of oxidative stress (Liang et al., 2017). Studies have shown that SIRT5 overexpression in neuroblastoma (Liang et al., 2017) and cardiomyocytes (Liu et al., 2013) can reduce the level of oxidative stress caused by H_2_O_2_. Furthermore, SIRT5 can deacetylate SOD1, thus strengthening the antioxidant effect of SOD1 against ROS (Lin et al., 2013). SIRT5 may inhibit ferroptosis by reducing oxidative stress, which remains to be investigated yet. Undoubtedly, more studies are needed to demonstrate the correlations of SIRT4 and SIRT5 with ferroptosis.

Studies have reported that SIRT6 can also inhibit ferroptosis. Cai et al. found that SIRT6 silencing led to the inactivation of the Keap1/NRF2 pathway and downregulation of GPX4 expression, promoting ferroptosis in gastric cancer cells (Cai et al., 2021). In addition, SIRT6 can protect human mesenchymal stem cells against oxidative stress-associated damage by activating NRF2 (Pan et al., 2016). Wang’s team demonstrated that sodium hydrosulfide (NaHS) had anti-inflammatory effects and anti-ferroptosis effects by up-regulating SIRT6 in type 1 diabetic mouse model (Wang et al., 2021c). Recently, SIRT6 has been demonstrated to inhibit nuclear transcription of NF-κB and inactivate NF-κB to facilitate ferroptosis in pancreatic cancer, thereby exerting anti-tumor effects (Gong et al., 2022). Fang et al. (2022) demonstrated that SIRT6 overexpression could reverse the decreased levels of GPX4, SLC7A11, and GSH and reduce the cellular accumulation of MDA and ROS to inhibit ferroptosis, ultimately restoring bone formation and angiogenesis and relieving femoral head necrosis. Mi et al. demonstrated that melatonin inhibited ferroptosis in rat lens epithelial cells through SIRT6/p-NRF2/GPX4 and SIRT6/NCOA4/FTH1 pathways, neutralized lipid peroxidation toxicity, and delayed the formation of cataract caused by ultraviolet rays exposure (Mi et al., 2023). In addition to direct evidence, SIRT6 can also modulate ferroptosis-associated genes, which indirectly demonstrates that SIRT6 can regulate ferroptosis. SIRT6 deacetylates and destabilizes p53 (Ghosh et al., 2018), and, in turn, the transcription factor p53 increases the expression of miR-34a that represses SIRT6 (Lefort et al., 2013). The regulation of SIRT6 by miR-34a may form a positive feedback loop for the p53 function. Moreover, it has been elucidated that SIRT6 can reduce the level of oxidative stress by activating the AMPK-FOXO3a axis, thereby protecting cardiomyocytes from ischemia-reperfusion injury (Wang et al., 2016). Pan et al. (2016) found that SIRT6 also co-activated NRF2 and its downstream antioxidant factors to protect mesenchymal stem cells from oxidative stress.

In chronic renal diseases caused by hypertension, SIRT7 can alleviate renal ferroptosis and epithelial-mesenchymal transition in hypertensive states by promoting the KLF15/NRF2 signaling pathway, thereby reducing renal fibrosis, injury, and dysfunction (Li et al., 2022). SIRT7 can regulate ferroptosis-associated mitochondrial ferritin expression and p53 activity. Carles Díez-López conducted genome-wide and TaqMan^®^ low-density array analyses and determined low SIRT7 and mitochondrial ferritin levels in patients with chronic heart failure and systemic iron deficiency; they also demonstrated that low-to-moderate levels of SIRT7 and mitochondrial ferritin were associated with an increased risk of all-cause mortality and admission to hospital with heart failure (Diez-Lopez et al., 2021). The function of SIRT7 to deacetylate p53 remains controversial. Vakhrusheva et al. found increased levels of acetylated K382 on p53 in SIRT7-knockout mice, and *in vitro* experiments also proved that the deacetylase activity of SIRT7 was comparable to that of SIRT1 with the p53 peptide acetylated at K382 as a substrate (Vakhrusheva et al., 2008). However, Barber et al. revealed that SIRT7 could not deacetylate p53-K382 either *in vitro* or *in vivo* (Barber et al., 2012). Likewise, Michishita et al. demonstrated that SIRT7 purified from Hi5 insect cells also failed to deacetylate p53-K382 (Michishita et al., 2005).

**FIGURE 2:**
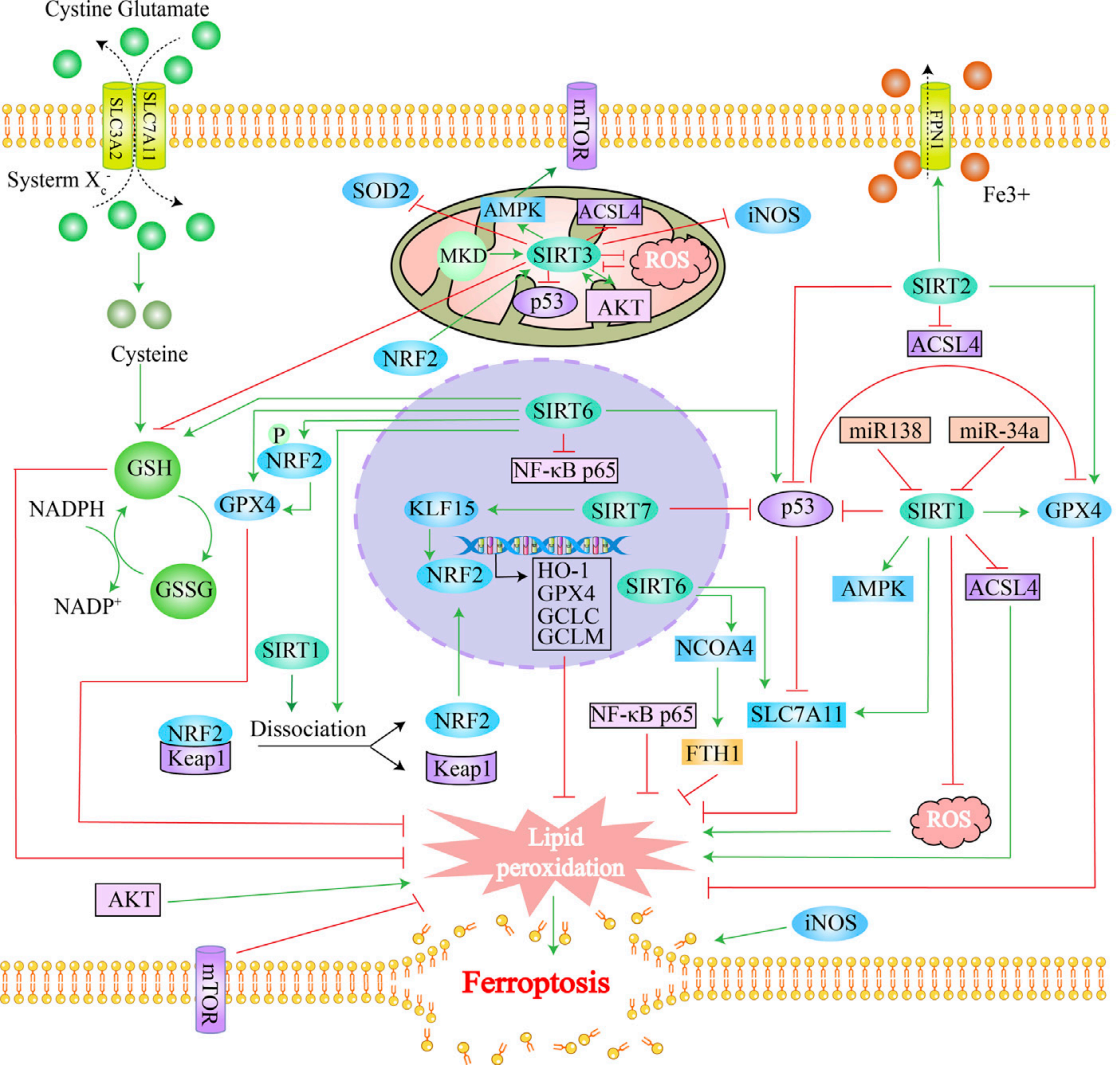
Overview of sirtuins and ferroptosis: Green lines represent promotion effects and red lines represent inhibition effects in the figure. We constructed sirtuins family pathways related to ferroptosis using Adobe illustrator software according to the gene pathways described in the text. Schematic diagram illustrating the distribution of sirtuins family members in the cell, their regulatory mechanisms, as well as functions. Sirtuins regulate ferroptosis by mediating lipid peroxides, ROS levels as well as iron content through different molecular pathways.

Collectively, the family members of sirtuins (deacetylases) are all implicated in varying degrees in the regulation of the ferroptosis process, and the regulatory roles of the sirtuins family in ferroptosis, the molecular mechanisms, and the diseases involved are summarized in Table 2 and Figure 2.

**TABLE 2:** The regulatory roles, molecular mechanisms, and research background of sirtuins in ferroptosis.

Gene	Signal pathway	Effect on ferroptosis	Mechanism	Background	Ref
SIRT1	USP22-SIRT1-p53/SLC7A11	inhibit	USP22 inhibited ferroptosis via SIRT1-p53/SLC7A11 pathway	cardiomyocyte	Ma et al. (2020)
	SIRT1-p53/ACSL4-GPX4/SLC7A11	inhibit	Overexpression of SIRT1 inhibited lipid peroxidation and diminished MDA levels, reversed SLC7A11 and GPX4 downregulation, ACSL4 and acetylated p53 upregulation to inhibit ferroptosis	lung epithelial cells	Chen et al. (2022b)
	MiR-138-5p-SIRT1/NRF2	inhibit	MiR-138-5p inhibited the activity of SIRT1 and NRF2 to promote ferroptosis	diabetic retinopathy	Tang et al. (2022)
	SIRT1-NRF2-HO-1/GPX-4	inhibit	Edaravone ameliorated ferroptosis via SIRT1/NRF2/HO-1/GPX-4 pathway	depressive and anxiety-like behaviors	Dang et al. (2022)
	SIRT1/NRF2	inhibit	Fisetin attenuated cardiomyopathy by inhibiting ferroptosis through SIRT1/NRF2 signaling pathway activation	cardiomyopathy	Li et al. (2021)
	SIRT1/NRF2/HO-1	inhibit	Ulinastatin protected against liver injury by alleviating ferroptosis via the SIRT1/NRF2/HO-1 pathway	liver injury	Wang et al. (2021b)
	SIRT1/NRF2/HO-1	Inhibit	Activation of SIRT1/NRF2/HO-1 signaling pathway inhibited ferroptosis	hippocampus	Liu et al. (2022a)
	SIRT1	promote	Intestinal SIRT1 deficiency protected mice by mitigating ferroptosis	liver injury	Zhou et al. (2020b)
	SIRT1	promote	Inhibiting the expression and activity of SIRT1 can inhibit ferroptosis	head and neck cancer	Lee et al. (2020)
	SIRT1	promote	BRD4 inhibitor (+)-JQ1 induced ferroptosis by enhancing the expression of SIRT1	cancer	Sui et al. (2019)
	MiR-34a-5p/SIRT1	inhibit	MiR-34a-5p inhibited the expression of SIRT1 to induce ferroptosis	PC12 cells	Hao et al. (2022)
	SIRT1/GPX4	inhibit	Calorie restriction protected against contrast-induced nephropathy via SIRT1/GPX4 activation	nephropathy	Fang et al. (2021)
	SIRT1-autophagy axis	inhibit	SIRT1 inhibited the ferroptosis of foam cells in excess iron by autophagy	atherosclerosis	Su et al. (2021)
	SIRT1-autophagy axis	inhibit	SIRT1-autophagy axis inhibited oxidative stress-induced ferroptosis	human nucleus pulposus cells	Zhou and Ruan (2022)
	SIRT1/NRF2	inhibit	Activating SIRT1 and upregulating the expression of NRF2 can against ferroptosis	sepsis-induced cardiomyopathy	Zeng et al. (2023)
	SIRT1	inhibit	Mitochondrial damage resulted from SIRT1 inactivation plays an important role in ferroptosis	schwan cells	Liang et al. (2023)
	SIRT1/SLC7A11	inhibit	PUM2 promoted ferroptosis by inhibiting SIRT1/SLC7A11	neuroinflamma-tion and brain damage	Liu et al. (2023)
	SIRT1	promote	Sodium fluoride upregulated SIRT1 and promoted ferroptosis	liver in chicken	Wang et al. (2023)
SIRT2	SIRT2-FPN1/GPX4/ACSL4	inhibit	Overexpression of SIRT2 inhibited ferroptosis by regulating FPN1, GPX4, ACSL4, iron accumulation and oxidant stress	neuropathic pain	Zhang et al. (2022c)
	SIRT2-P53	inhibit	SIRT2 inhibition exacerbated p53-mediated ferroptosis	traumatic brain injury	Gao et al. (2021)
SIRT3	SIRT3-P53	inhibit	SIRT3 deacetylation p53 to inhibit ferroptosis induced by ROS.	osteosarcoma	Jin et al. (2021)
	SIRT3	Inhibit	Knocking down SIRT3 inhibited ferroptosis to reduce oligodendrocytes survival	oligodendrocytes	Novgorodov et al. (2018)
	MKD-SIRT3	inhibit	MKD inhibited ferroptosis maybe by promoting the expression of SIRT3	cognitive deficiency	Wang et al. (2022)
	SIRT3- AMPK-mTOR/SIRT3-GPX4	promote	SIRT3 deficiency inhibited the AMPK/mTOR pathway and promoted GPX4 levels to suppress ferroptosis	gestational diabetes mellitus	Han et al. (2020)
	SIRT3-AKT	promote	Silence of SIRT3 gene inhibited AKT-dependent ferroptosis	gallbladder cancer	Liu et al. (2021b)
	AKT-SIRT3/SOD2	inhibit	LCZ696 increased the expression of SIRT3 and deacetylated its target gene SOD2 by activating AKT, thereby inhibiting ferroptosis	cardiac toxicity	Liu et al. (2022b)
	NRF2/SIRT3/iNOS	inhibit	Inactivating the NRF2/SIRT3 signaling pathway through ROS, thereby promoting the expression of iNOS, ultimately leading to ferroptosis	bronchial epithelial cells	Zi et al. (2023)
	SIRT3/AMPK/mTOR	inhibit	Activating the SIRT3/AMPK/mTOR pathway can inhibit ferroptosis	polycystic ovary syndrome	Peng et al. (2023)
SIRT6	SIRT6-Keap1/NRF2/GPX4	promote	SIRT6 silencing inactived Keap1/NRF2 signalling pathway and suppressed GPX4 to drive ferroptosis	gastric cancer	Cai et al. (2021)
	SIRT6	inhibit	Sodium hydrosulfide (NaHS) upregulated the expression and activity of SIRT6 to suppress ferroptosis	type 1 diabetic	Wang et al. (2021c)
	SIRT6-NF-κB	promote	SIRT6 promoted ferroptosis in pancreatic cancer by regulating the expression NF-κB	pancreatic cancer	Gong et al. (2022)
	SIRT6-SLC7A11/GPX4/GSH	inhibit	SIRT6 overexpression could reverse the decrease of GPX4, SLC7A11, and GSH and reduce MDA and ROS to inhibit ferroptosis	glucocorticoid-Induced osteonecrosis	Fang et al. (2022)
	SIRT6/p-NRF2/GPX4 and SIRT6/NCOA4/FTH1	inhibit	SIRT6/p-NRF2/GPX4 and SIRT6/NCOA4/FTH1 can inhibit ferroptosis	rat lens epithelial cells	Mi et al. (2023)
SIRT7	SIRT7/KLF15/NRF2	inhibit	SIRT7 can alleviate renal ferroptosis by promoting the KLF15/NRF2 signaling pathway	chronic renal diseases	Li et al. (2022)

## 3 Implications of bioinformatic analysis

**FIGURE 3:**
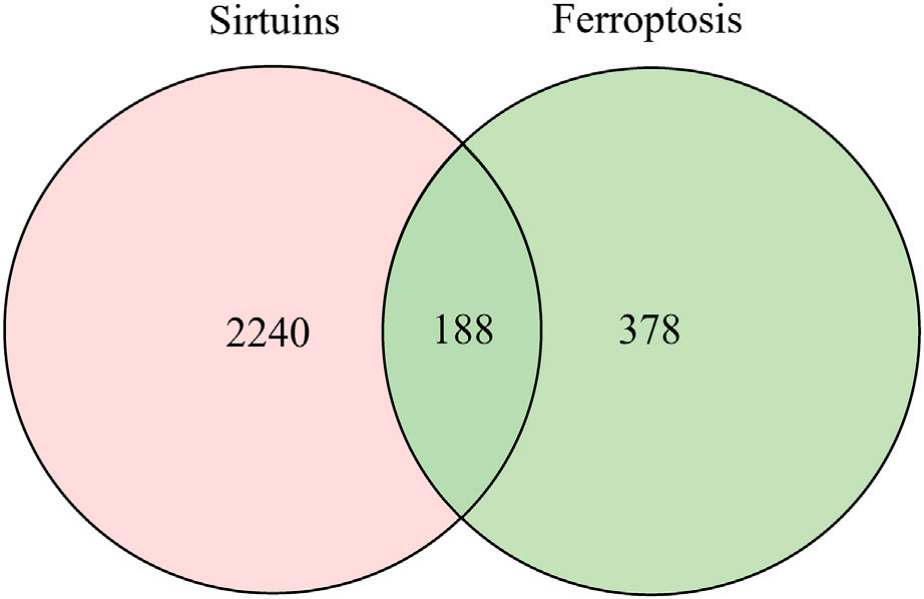
Venn diagram of downstream regulatory genes in sirtuins and ferroptosis: After screening the sirtuins family-related genes and ferroptosis-related genes from the Genecards and Ferrdb databases, respectively, a Venn diagram of the genes linked to the seven members of the sirtuins family and the genes related to ferroptosis was constructed in the Evenn webpage.

**FIGURE 4:**
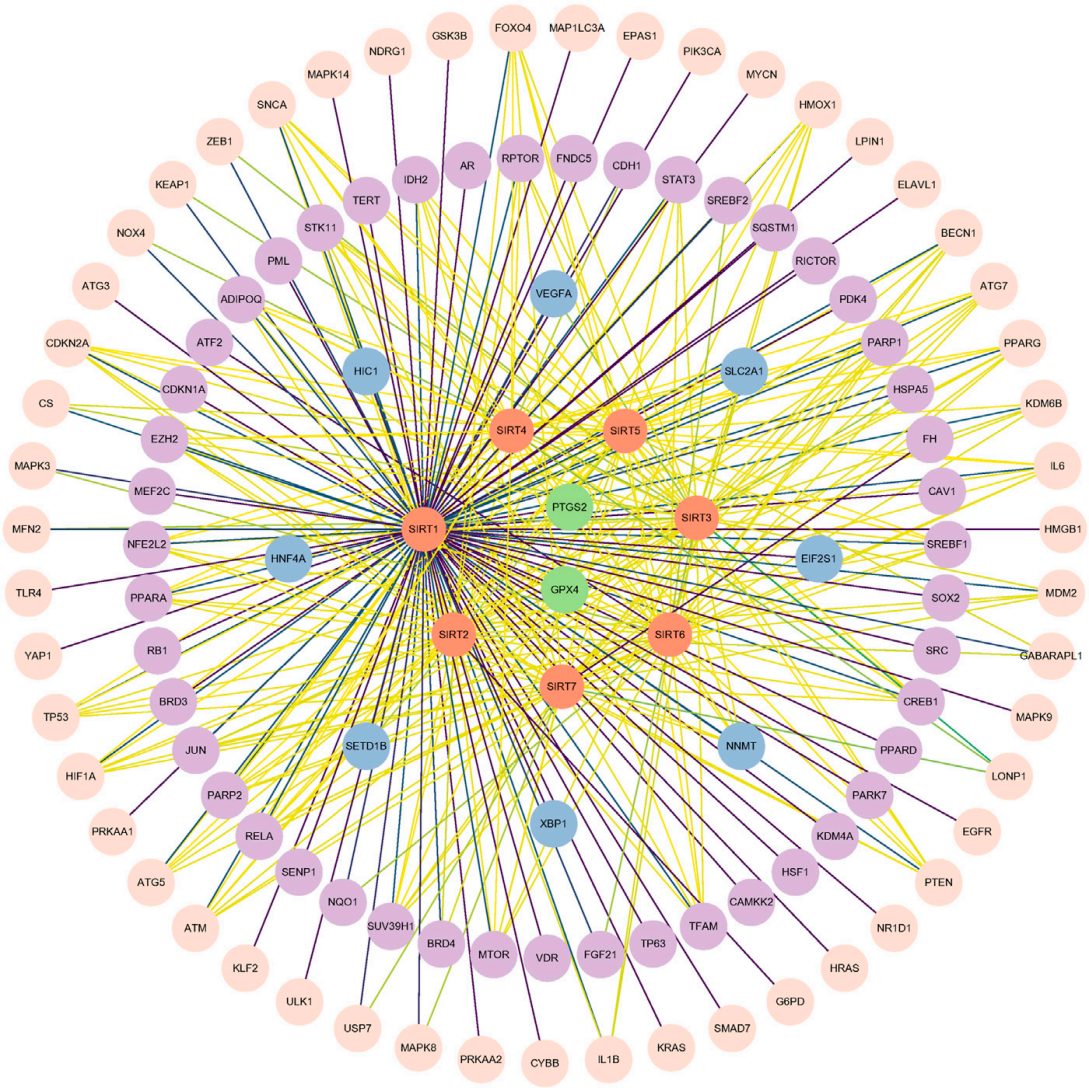
The interaction data with humans as species were derived from STRING (http://string-db.org, accessed 17 March 2023) based on the intersection in the Venn diagram (Figure 3), and this map shows the direct interaction between seven members in the sirtuins family and proteins involved in ferroptosis after data processing with Cytoscape tool. Orange circles denote sirtuins members, and other circles denote ferroptosis-associated genes. The lines (yellow-green-purple) indicate the gradual enhancement of protein-protein interaction.

To more clearly and intuitively view the pathways involved in ferroptosis mediated by sirtuins, the genes interacting with 7 sirtuins family members and ferroptosis-associated genes were screened from Genecards and Ferrdb (http://www.zhounan.org/ferrdb/) databases, respectively; then a Venn diagram of sirtuins-related genes and ferroptosis-related genes was plotted in the Evenn website (http://www.ehbio.com/test/venn/#/) as depicted in Figure 3 and Supplementary Table S1. From this Venn diagram, 188 potential interaction targets were found in the intersection. Among these potential targets, MTOR, TP53, NFE2L2, HIF1A, HMOX1, G6PD and KEAP1 were mentioned in the text, while the remaining genes are to be investigated. To gain more insight into the relationship between these 188 genes and sirtuins, the overlapping genes in the above Venn diagram were imported into the STRING database and a protein-protein interaction (PPI) network was constructed. The potential key direct action targets were screened using the Cytoscape tool. A total of 115 nodes and 324 lines are shown in Figure 4. The results showed a degree 30 for all sirtuins, a degree of 104 for SIRT1, a degree of 37 for SIRT2, a degree of 44 for SIRT3, a degree of 32 for SIRT4, a degree of 35 for SIRT5, a degree of 38 for SIRT6, and a degree of 34 for SIRT7. The aforesaid findings illustrated that sirtuins were strongly linked to multiple ferroptosis-associated genes. We listed multiple ferroptosis-associated genes interacting with sirtuins in the Supplementary Table S1, and more mechanisms of sirtuins regulating ferroptosis-related genes have not been identified, which is our future research direction.

## 4 Perspectives

Ferroptosis is a lipid peroxidative damage caused by overload free iron. Ferroptosis has been found to occur in a variety of diseases, including cardiovascular diseases, tumors, liver diseases, kidney diseases, neurological diseases, metabolic diseases, respiratory diseases (such as the global pandemic COVID-19) and immune system diseases. With the in-depth study of the mechanism of ferroptosis, targeted drugs have been developed rapidly. These drugs can mainly be divided into several categories, including targeting the Xc system (such as piperazine erastin), GPX4 (such as altretamine), iron metabolism pathway (such as dihydroartemisinin), lipid metabolism pathway (such as Zileuton) and other metabolic pathways (such as NADH, NADPH). Although there are a variety of drugs targeting ferroptosis, the treatment might be limited by potential side effects. Sirtuins-specific drugs have been developed, some of which have already been applied in clinical trials. In addition, we have predicted 188 target genes of sirtuins associated with ferroptosis through bioinformatics analysis, most of which have not been reported yet. It is worth mentioning that the in-depth study of these pathways may provide targets and a theoretical basis for the development of ferroptosis-targeted drugs.

Current researches related to ferroptosis mechanisms mainly focus on lipid metabolism, ROS, and iron metabolism. With a deeper understanding of ferroptosis and the sirtuins family, researchers are increasingly aware of the link between sirtuins and ferroptosis. Of particular importance, the homeostasis of both lipid ROS and iron metabolism levels can be regulated by sirtuins target genes. For instance, p53 is an important ferroptosis trigger, and NRF2 is a key antioxidant gene, both of which can be regulated by SIRT1 (Ma, 2013; De Angelis et al., 2015; Bellezza et al., 2018). Mitochondria, the main organelle for producing ROS, are multifaceted regulators of ferroptosis. How important the role of SIRT3, SIRT6 and SIRT7, which are located in mitochondria, plays in scavenging ROS and the related ferroptosis remains to be elucidated. In addition, current studies on the role of sirtuins in ferroptosis are limited in the context of diseases, while their functions in ontogenesis have not been reported. The exploration of these fields may help researchers better understand the mechanism of sirtuins in ferroptosis.
