# Supplementary material for: The roles of sirtuins in ferroptosis
Source: Front Physiol. 2023 Apr 20;14:1131201. doi: 10.3389/fphys.2023.1131201 (PMC10157232; doi:10.3389/fphys.2023.1131201)
Supplement: Supplementary file 1 [file Table1.DOCX]

**Supplement Table 1.** Intersection genes of sirtuins-related genes and ferroptosis-related genes

| gene | HGNC | gene | HGNC | gene | HGNC | gene | HGNC |
| --- | --- | --- | --- | --- | --- | --- | --- |
| TP53 | 11998 | PML | 9113 | EGR1 | 3238 | ARF6 | 659 |
| PARP1 | 270 | RPTOR | 30287 | SOX2 | 11195 | UBC | 12468 |
| NFE2L2 | 7782 | KLF2 | 6347 | CD44 | 1681 | MTDH | 29608 |
| HIF1A | 4910 | RB1 | 9884 | NQO1 | 2874 | KEAP1 | 23177 |
| RELA | 9955 | USP7 | 12630 | H19 | 4713 | MYB | 7545 |
| MTOR | 3942 | FBXW7 | 16712 | VCP | 12666 | RRM2 | 10452 |
| PRKAA1 | 9376 | STK11 | 11389 | DLD | 2898 | SQSTM1 | 11280 |
| HMGB1 | 4983 | TGFB1 | 11766 | P4HB | 8548 | AQP3 | 636 |
| EPAS1 | 3374 | ATG7 | 16935 | MEF2C | 6996 | KDM6B | 29012 |
| SREBF1 | 11289 | MIR212 | 31589 | PGD | 8891 | ACO1 | 117 |
| STAT3 | 11364 | IDH2 | 5383 | SP1 | 11205 | KDM4A | 22978 |
| AR | 644 | HSF1 | 5224 | PARP2 | 272 | NR5A2 | 7984 |
| MAPK1 | 6871 | AURKA | 11393 | RICTOR | 28611 | SENP1 | 17927 |
| SNCA | 11138 | CDKN2A | 1787 | GABARAPL1 | 4068 | SLC7A5 | 11063 |
| PPARA | 9232 | FZD7 | 4045 | RPL8 | 10368 | AGPS | 327 |
| JUN | 6204 | YAP1 | 16262 | CPEB1 | 21744 | EIF2S1 | 3265 |
| PRKAA2 | 9377 | IL6 | 6018 | YTHDC2 | 24721 | PGRMC1 | 16090 |
| HIC1 | 4909 | ATF4 | 786 | NRAS | 7989 | TRIB3 | 16228 |
| VEGFA | 12680 | TFAM | 11741 | AKR1C1 | 384 | ETV4 | 3493 |
| MAPK8 | 6881 | CS | 2422 | MAPKAP1 | 18752 | BRD3 | 1104 |
| KRAS | 6407 | HRAS | 5173 | PPP1R13L | 18838 | BRD7 | 14310 |
| ELAVL1 | 3312 | CDH1 | 1748 | FLT3 | 3765 | CCDC6 | 18782 |
| PPARG | 9236 | FOXO4 | 7139 | NEDD4 | 7727 | KIF20A | 9787 |
| ADIPOQ | 13633 | TLR4 | 11850 | TP63 | 15979 | NR1D2 | 7963 |
| PTEN | 9588 | EGFR | 3236 | CYBB | 2578 | SETD1B | 29187 |
| HNF4A | 5024 | PARK7 | 16369 | PRDX1 | 9352 | TSC22D3 | 3051 |
| ASNS | 753 | NOX4 | 7891 | MAP1LC3A | 6838 | METTL14 | 29330 |
| TERT | 11730 | MIR9-1 | 31641 | MFN2 | 16877 | ZFP36 | 12862 |
| CREB1 | 2345 | CDKN1A | 1784 | NCOA3 | 7670 | FNDC5 | 20240 |
| HSPA5 | 5238 | PPARD | 9235 | FH | 3700 | SMG9 | 25763 |
| HMOX1 | 5013 | NDRG1 | 7679 | GPX4 | 4556 | MEG3 | 14575 |
| MAPK3 | 6877 | VDR | 12679 | ACSL3 | 3570 | NEAT1 | 30815 |
| ZEB1 | 11642 | BECN1 | 1034 | ATG3 | 20962 | HOTAIR | 33510 |
| ULK1 | 12558 | SLC2A1 | 11005 | DCAF7 | 30915 | MIR135B | 31760 |
| NNMT | 7861 | DDIT3 | 2726 | MIR27A | 31613 | MIR30B | 31625 |
| CAMKK2 | 1470 | ATF3 | 785 | MIR761 | 37305 | MIR17 | 31547 |
| GSK3B | 4617 | SRC | 11283 | PIK3CA | 8975 | MIR424 | 31881 |
| LONP1 | 9479 | PTGS2 | 9605 | MAPK14 | 6876 | IFNG | 5438 |
| XBP1 | 12801 | CAV1 | 1527 | SNORA16A | 32605 | PARP16 | 26040 |
| SUV39H1 | 11479 | TIMP1 | 11820 | MAPK9 | 6886 | HSPB1 | 5246 |
| MDM2 | 6973 | ATG5 | 589 | BRD4 | 13575 | NR4A1 | 7980 |
| RIPK1 | 10019 | USP11 | 12609 | IL1B | 5992 | PRDX6 | 16753 |
| EZH2 | 3527 | G6PD | 4057 | NR1D1 | 7962 | LCN2 | 6526 |
| MMP13 | 7159 | LPIN1 | 13345 | HELLS | 4861 | SREBF2 | 11290 |
| MIR9-3 | 31646 | MYCN | 7559 | SOCS1 | 19383 | VDAC2 | 12672 |
| ATM | 795 | SMAD7 | 6773 | NOX1 | 7889 | HBA1 | 4823 |
| FGF21 | 3678 | ATF2 | 784 | PDK4 | 8812 | MLST8 | 24825 |
